# First-in-man application of Liwen RF™ ablation system in the treatment of drug-resistant hypertrophic obstructive cardiomyopathy

**DOI:** 10.3389/fcvm.2022.1028763

**Published:** 2022-11-09

**Authors:** Zihao Wang, Rong Zhao, Horst Sievert, Shengjun Ta, Jing Li, Stefan Bertog, Kerstin Piayda, Mengyao Zhou, Changhui Lei, Xiaojuan Li, Jiani Liu, Bo Xu, Bo Feng, Rui Hu, Liwen Liu

**Affiliations:** ^1^Xijing Hypertrophic Cardiomyopathy Center, Department of Ultrasound, Xijing Hospital, Fourth Military Medical University, Xi’an, China; ^2^Xijing Hypertrophic Cardiomyopathy Center, Department of Cardiac Surgery, Xijing Hospital, Fourth Military Medical University, Xi’an, China; ^3^CardioVascular Center, Frankfurt, Germany; ^4^Minneapolis Veterans Affairs Medical Center, Minneapolis, MN, United States

**Keywords:** hypertrophic obstructive cardiomyopathy, percutaneous intramyocardial septal radiofrequency ablation, radiofrequency ablation system, conformal ablation, first-in-man application, Liwen RF

## Abstract

**Objectives:**

This study sought to evaluate the clinical applicability of the Liwen Liu RF™ ablation system for percutaneous intramyocardial septal radiofrequency ablation (PIMSRA).

**Background:**

Data on new cardiac radiofrequency ablation devices for the treatment of hypertrophic obstructive cardiomyopathy (HOCM) are limited.

**Materials and methods:**

From July 2019 to July 2020, a total of 68 patients with drug-resistant HOCM, who underwent PIMSRA with the Liwen RF™ ablation system, which has an ablation electrode of stepless adjustable length, were prospectively enrolled. Safety endpoints included, amongst others, the occurrence of pericardial effusion and/or hemorrhage, cardiac arrhythmias, device failure and procedural death. The reduction in left ventricular outflow tract (LVOT) gradients at 12 months follow-up were used as a surrogate marker for device efficacy.

**Results:**

All procedures were technically successful. The total energy output time of the system was 75.8 (IQR: 30.0) min, and the average power was 43.61 ± 13.34 watts. No ablation system error occurred. The incidence of pericardial effusion or hemorrhage, transient arrhythmia and resuscitation was 8.8, 39.7, and 1.5% during procedure, respectively. None of the patients died. During 30-day follow-up, there were no complications with the exception of a pericardial effusion in one patient (1.5%). No further complications were reported after 30 days. The patients’ resting [baseline: 75 (IQR: 48) *vs.* 12-months: 12 (IQR: 19) mmHg, *p* < 0.001] and provoked [baseline: 122 (IQR: 53) *vs.* 12-months: 41 (IQR: 59) mmHg, *p* < 0.001] LVOT gradients decreased significantly during follow-up.

**Conclusion:**

In this study, we demonstrate the safety and feasibility of the Liwen RF™ ablation system to treat HOCM. The system allows for significant and sustainable LVOT gradient reduction during 12-months of follow-up. Hence, the Liwen RF™ ablation system is a promising new device that has the potential to become an alternative to existing septal reduction concepts in HOCM patients.

## Introduction

Hypertrophic cardiomyopathy is a common hereditary cardiomyopathy, with an incidence of about 2–5 per 1,000 in the adult population (1–3). Asymmetrical left ventricular hypertrophy is the main manifestation and cannot be explained by other cardiac diseases (4, 5). About two-thirds of patients have resting and/or provoked left ventricular outflow tract (LVOT) obstruction, known as hypertrophic obstructive cardiomyopathy (HOCM) (6, 7) which is associated with increased morbidity and limited life-expectancy (1, 8, 9). For HOCM patients with significant symptoms that do not respond to medical treatment, septal reduction therapies are a treatment option. Surgical myectomy (SM) is considered to be the gold standard (10, 11), and alcohol septal ablation (ASA) a less invasive alternative (12). There is evidence that in specialized high-volume centers, the LVOT gradient of patients after SM is generally less than 10 mmHg, and over 90% of patients experience long-term relief of symptoms after surgery (13, 14). Likewise, in an experienced ASA team, nearly 95% of treated patients had a gradient reduction of at least 50% as compared to baseline, with symptom improvement and in-hospital mortality less than 1% (15, 16).

In recent years, the development of percutaneous intramyocardial septal radiofrequency ablation (PIMSRA, Liwen procedure™) has emerged as a new option for the interventional treatment of HOCM (17). Exploratory results in a small cohort of patients, who could not tolerate thoracotomy and did not choose ASA because of contraindications or unacceptable risks, with a strong willingness to accept minimally invasive treatment, showed a good safety profile and significant and sustainable LVOT gradient reduction (18). Additionally, the feasibility of PIMSRA combined with transcatheter aortic valve replacement (TAVR) for aortic stenosis with LVOT obstruction was demonstrated in a case report (19). However, the radiofrequency needle electrode of the Cool-tip system used in previous trials cannot match the intended ablation range perfectly. We, therefore, developed a new radiofrequency ablation system, the Liwen RF™ system (Hangzhou Nuo Cheng Medical Instrument Co., Ltd., Hangzhou, Zhejiang, China), with an ablation electrode of stepless adjustable length, to achieve adequate and accurate ablation at different anatomical sites of interventricular septum (IVS), referred to as “conformal ablation”. Different from the emphasis on tumor ablation that “tumor tissues of different shapes and adjacent normal tissues should be included as much as possible”, conformal ablation for HOCM requires strict control of ablation scope within the IVS to avoid perforation and ensure a safe distance between ablation boundary and endocardium to protect the conduction system from damage. This is achieved by adjusting the exposure length of the needle electrode, which we call the “Working section length”. This study aims to prove that the Liwen RF ablation system can be used safely and effectively in the treatment of drug-resistant HOCM.

## Materials and methods

### Patients

This is an open label, one-arm, prospective, non-randomized study. Patients with HOCM and severe LVOT obstruction and refractory symptoms despite adequate medication were eligible for trial participation. Treatment options were discussed with the patient, and cases were presented in a multi-disciplinary heart team. Patients provided written informed consent. Table 1 lists the inclusion and exclusion criteria for this study. Patients with a higher sudden cardiac death index (SCDI) were thought to be more likely to have an accident during ablation, so SCDI ≥ 10 was considered a contraindication or patient exclusion criterion.

**TABLE 1:** Inclusion and exclusion criteria of patients.

**Inclusion criteria:**
(1) Age between 18 and 70
(2) Resting or provoked LVOT gradient ≥ 50 mmHg
(3) Septal thickness of at least 15 mm
(4) Refractoriness to medical therapy with a beta-blocker, and/or CCB
(5) Symptoms attributed to HOCM
(6) NYHA functional class ≥ II
(7) Informed consent and agreement to complete follow-up
**Exclusion criteria:**
(1) Pregnancy or breast-feeding
(2) Non-obstructive hypertrophic cardiomyopathy
(3) Septal thickness ≥ 30 mm
(4) SCDI ≥ 10
(5) Presence of concomitant heart disease requiring surgery
(6) Symptomatic heart failure at rest despite maximal guideline directed medical therapy and LVEF < 40%

CCB, calcium channel blocker; HOCM, hypertrophic obstructive cardiomyopathy; LVEF, left ventricular ejection fractions; NYHA, New York Heart Association; SCDI, sudden cardiac death index.

The study was registered at chictr.org (ChiCTR2000031936) and was conducted in accordance with the ethical standards of the Helsinki Declaration. The protocol was approved by the Ethics Committee of Xijing Hospital (KY-20192076-F-1).

### Equipment

**The Liwen RF ablation system consists of the following components:**

– Liwen RF ablation electrode kit (RFET02A): sterile packaged for single use. As the core device of the Liwen RF system, the ablation electrode is also the most important part of the equipment. It has been specifically developed for radiofrequency ablation of the ventricular septal myocardium.
– Liwen RF ablation generator (RFGR100): Reuse unit. Used to generate radiofrequency energy and output to the electrode needle. The generator incorporates an algorithm designed for cardiac ablation that will simultaneously detect the impedance and temperature of the electrode needle for energy output management. The equipment is operated by an engineer.
– System cooling pump (RFPP01): Reuse unit. This is used to cool the electrode needle and prevent overheating in the ablation area. The device should be kept on during the energy output. The refrigerant is sterile water placed in an ice bucket during procedure.
– Patient circuit electrode: Disposable non-sterile consumables. It is used to form a loop with the ablation electrode and the patient’s tissue which is connected to the generator by a cable attached to the ablation electrode kit.

**FIGURE 1:**
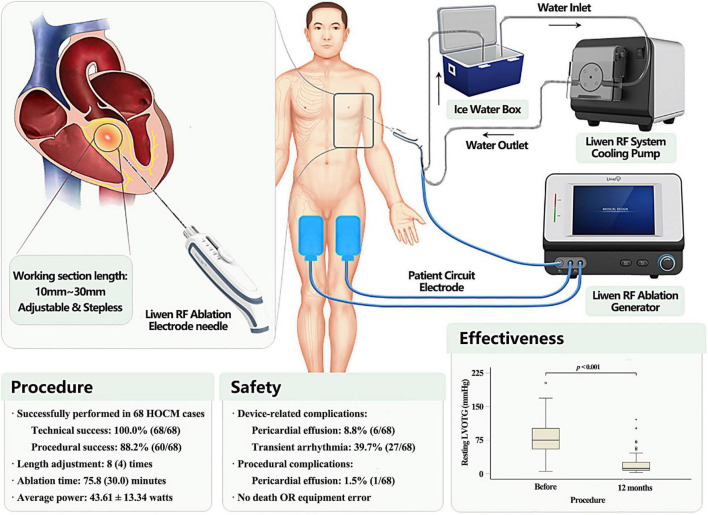
Description of Liwen RF™ ablation system and main findings. Liwen RF™ (Hangzhou Nuo Cheng Medical Instrument Co., Ltd., Hangzhou, Zhejiang, China) is a novel radiofrequency ablation system with a stepless adjustable length needle electrodes for percutaneous intramyocardial septal radiofrequency ablation (PIMSRA). The device is an integrated system, comprising ablation electrode kit, ablation generator, cooling system, and patient circuit.

The Liwen RF ablation electrode kit must be used in conjunction with other parts of the Liwen RF ablation system for PIMSRA. It mainly includes the following items: puncture needle (17G), electrode needle (18G), inflow tube, outflow tube, needle tube positioning parts and negative plate connecting cable. It should be noted that except for the patient circuit electrode connection cable, all other items are sterile. The exposed length of the needle electrode, that is, the working section length of Liwen RF, can be adjusted freely within 10∼30 mm by the slider on the handle. As shown in Figure 1, five scales are displayed, corresponding to the length of exposed electrode in increments of 5 mm. Nevertheless, in the actual adjustment process, the control of working section length is stepless. The longer the working section length is, the greater the ablation area length, width and thickness will be. Therefore, with the help of imaging equipment, the exposed length of the needle electrode and its position in the IVS should be adjusted according to the degree of cardiac hypertrophy and the desired ablation range.

Before ablation begins, the electrode needle detects the initial impedance of the surrounding tissue. According to our data, the impedance of the septal tissue before ablation ranged from 92 to 115 ohms, with a mean impedance of 103.20 ± 9.13 ohms. With the progress of ablation, the impedance decreased slowly at first, and then gradually increased after a plateau. When the impedance rises sharply to 150% of the initial value, the system enters a “Hibernation state”, at which time the system will suspend energy output for 15 s. After that, the system restarts and repeats the process of impedance detection until the next hibernation. Frequent hibernation indicates that the local tissue has been sufficiently ablated. In our experience, the number of cycles for ablation and hibernation is typically three.

### Assessments

Philips EPIQ 7C (Philips Medical Systems, Bothell, WA, USA) ultrasound imaging system was used for transthoracic echocardiography (TTE). Septal thickness, LVOT gradient, left ventricular ejection fractions (LVEF), and systolic anterior motion (SAM) of the mitral valve were assessed. SAM is classified into four grades on M-mode echocardiography. Grade 0: No systolic anterior motion of the mitral valve; Grade 1: Brief systolic anterior motion without septal contact; Grade 2: Systolic anterior motion with septal contact lasting < 1/3 of the systolic period; Grade 3: Systolic anterior motion with septal contact lasting ≥ 1/3 of the systolic period (20). Continuous wave doppler guided by color signal was used to measure the LVOT gradient (21, 22). The gradient under exercise stress was provoked by supine cycling (Semi-recumbent and tilting bicycle Ergometer, Lode BV, Groningen, Netherlands) and measured according to a standardized protocol (23). All sections and measurements were obtained in accordance with the recommendations of the American Society of Echocardiography for HOCM (24). The results of genetic tests were interpreted for pathogenicity according to the guidelines of the American College of Medical Genetics and Genomics (25).

### Procedure

The use of Liwen RF can be described through four workflow steps: device deployment, puncture, ablation, and withdrawal. Setup and removal of the device are performed jointly by the engineer and the physician. The only conductor on the needle, the working section, releases a high-frequency current in the myocardium which excites ions to generate heat. The local tissue temperature recorded by the electrode tip can reach above 80°C. As a result, the tissue become dehydrated, coagulates, and areas of necrosis are formed. At the same time, the occlusion of the surrounding vessels blocks the blood supply of the hypertrophic myocardium. All procedures follow the requirements of PIMSRA (18, 26). Temporary pacemaker placement is required for patients with pre-procedural right bundle branch block or left bundle branch block. During the procedure, which is performed in general anesthesia, the patient is placed on the left side at 30 to 45 degrees. Then ECG and Liwen RF system are connected. Under echocardiography guidance, the puncture needle is inserted through the guide frame, and reaches the front end of the area, which is thought to be ablated. Then, the needle is pulled out of the core and the electrode needle inserted into the body and pushed to the target area under echocardiography guidance. The ablation generator is turned on. The initial power is 20 watts and gradually increased to 70∼80 watts at 10∼20 watts increments. Ablation continues for 10 to 12 min. After adjusting the working section length and position of the needle electrode, the aforementioned steps are repeated. The number and location of the ablations are tailored to the expected ablation range. Dehydrated, coagulated tissue that has been completely ablated appears as a hyperechoic area on TTE. Ablation was considered satisfactory when the area was 30∼40 mm along both the long and short axes of the IVS and the thickness reached 2/3 of it. At this point, as shown in Figure 1, 3∼5 mm of unablated area must be maintained bilaterally with respect to the endocardium. If prolonged heart block or tachyarrhythmias were detected by ECG, ablation was suspended until normal rhythm was restored spontaneously or after lidocaine treatment. After all ablations are complete, the electrode needle is removed and pressure to the puncture point applied for 5–10 min. Vital signs were closely monitored for at least 15 min, during which TTE was performed to assess the range of ablation, cardiac structure, and hemodynamics. If the rhythm and hemodynamics were stable, the patients were transferred to the ICU for at least 24 h of monitor and recovery.

### Process monitoring and energy calculation

Throughout the procedure, an engineer continuously monitors system performance and records equipment errors. The ablation generator, the host of the system, has a touchable display for setting and viewing device parameters. According to the procedure situation, the engineer records the energy output time and maximum power of each ablation, and feedback to the operator. After the procedure, the generator calculates the average power of the entire ablation process. The average power and the ablation time are multiplied to obtain the total output energy.

### Endpoints

The primary endpoint is the occurrence of major adverse events (MAE) within 30 days. It is defined as any device or procedure related complication, including but not limited to death, emergency surgery, severe cardiac tamponade requiring pericardiocentesis or surgery, bleeding, and procedure-related stroke. We define device-related and procedural complications as follows: complications that occur while the ablation needle is in the body are considered device-related complications. After the device is removed, if new complications occur or existing complications change, they are classified as procedural complications. Secondary endpoints include: (i) Technical success: the ablation needle enters the myocardium smoothly and leaves completely at the end, and the system has no error throughout the procedure; (ii) Procedural success: Reduction of the resting or provoked LVOT gradient to < 30 mmHg or by ≥ 50% 12 months after procedure.

### Statistical analysis

The Shapiro-Wilk test was used to evaluate the data set for normal distribution. Quantitative data of normal distribution were expressed as mean ± standard deviation (SD), and the paired-sample *t*-test was used for comparison. Non-normally distributed quantitative data were represented by medians and interquartile range (IQR), and the Wilcoxon signed rank test was used for comparison. Qualitative data were expressed as absolute counts and percentages of total, and compared using the Pearson χ^2^ test. A *P*-value < 0.05 was considered statistically significant. The analyses were performed with SPSS software 26.0 (SPSS Inc., Chicago, IL, USA).

## Results

### Baseline characteristics

A total of 68 patients were consecutively enrolled from July 2019 to July 2020. During the study, all patients were treated with the same equipment. Baseline characteristics are summarized in Table 2. The average age was 47.74 ± 13.85 years, and 29.4% (20/68) were female. According to the prediction model (1), the overall risk of sudden cardiac death was intermediate to high because a median SCDI of ≥ 4% was present. Among the 68 patients, the IVS thickness was 23.56 ± 4.55 mm, and the resting LVOT gradient was 78 ± 39 mmHg, accompanied by typical clinical symptoms. All patients underwent genetic testing before the procedure. A total of 31 patients were identified with pathogenic variants in sarcomere protein and related genes, while the genetic profile of the remaining 37 cases has not yet been completely elucidated. Among patients with definite mutations, the proportion of myosin heavy chain (MYH7) gene mutation was 51.6% (16/31), and that of myosin binding protein C (MYBPC3) gene was 38.7% (12/31). Interestingly, one patient had a dual mutation of both MYBPC3 and MYH7. Other pathogenic variants include genes encoding cardiac troponin I (TNNI3) and the protein tyrosine phosphatase non-receptor-type 11 (PTPN11).

**TABLE 2:** Baseline characteristics of patients.

Group	Value
Age (years)-mean ± SD	47.74 ± 13.85
Female-n (%)	20 (29.4)
BSA (m^2^)-mean ± SD	1.80 ± 0.19
SCDI (%)-median (IQR)	4.28 (3.67)
**Complicating disease-n (%)**	
Hypertension	18 (26.5)
Coronary heart disease	4 (5.9)
Type 2 diabetes mellitus	4 (5.9)
**Gene mutation site-n (%)**	
MYH7	16 (23.5)
MYBPC3	12 (17.6)
TNNI3	1 (1.5)
PTPN11	1 (1.5)
MYBPC3 and MYH7*	1 (1.5)
Unknown sites	37 (54.4)
NYHA functional class ≥ III-n (%)	18 (26.5)
6-min walk test distance (m)-mean ± SD	451.5 ± 86.0
**Symptom-n (%)**	
Chest pain	66 (97.1)
Shortness of breath	54 (79.4)
Syncope/pre-syncope	31 (45.6)
LVEF (%)-median (IQR)	58 (4)
IVS (mm)-mean ± SD	23.56 ± 4.55
**Mitral valve SAM^†^-n (%)**	
Grade 0	5 (7.4)
Grade 1	2 (2.9)
Grade 2	30 (44.1)
Grade 3	31 (45.6)
Resting LVOTG (mmHg)-mean ± SD	78 ± 39
Provoked LVOTG (mmHg)-mean ± SD	126 ± 44

*Double mutation; ^†^Measured at rest. BSA, body surface area; IQR, interquartile range; IVS, interventricular septum; LVEF, left ventricular ejection fractions; LVOTG, left ventricular outflow tract gradient; NYHA, New York heart association functional class; SAM, systolic anterior motion; SCDI, sudden cardiac death index.

### Equipment feasibility and procedure data

According to our success definition, all 68 patients achieved satisfactory treatment results. The application success rate of Liwen RF ablation system in the treatment of drug-resistant HOCM was 100%. A series of procedure related parameters and possible technical errors are listed in Table 3. During the 68 procedures, the operator had to start/stop the ablation device 8 (IQR: 4) times to adjust the position and working section length to achieve the designed ablation range. The total energy output time was 75.8 (IQR: 30.0) min, and the cumulative energy release 186.02 (IQR: 114.97) kilojoules. The maximum and average power were 64 ± 18 and 43.61 ± 13.34 watts, respectively. There was no interruption or failure of the procedure caused by the error of the ablation system. Although we were fully prepared and formulated corresponding solutions and treatment plans, no error occurred during this period. The integrity of the ablation needle was routinely examined after the procedure. Needle fracture did not occur in any of the interventions. Interestingly, in 7 (10.3%) patients, the needles were bent after removal. The degree of bending varies from patient to patient and ranges from 5 to 60°. Further analysis showed that needle bending was not associated with complications or efficacy. Therefore, we do not consider it a type of equipment error.

**TABLE 3:** Data of equipment and procedure.

Group	Value
Technical success-n (%)	68 (100)
Procedural success-n (%)	60 (88.2)
Time of ablation (minutes)-median (IQR)	75.8 (30.0)
Ablation times-median (IQR)	8 (4)
**Range of ablation (mm)-median (IQR)**	
Length	40 (9)
Width	40 (13)
Thickness	16 (4)
Maximum power (W)-mean ± SD	64 ± 18
Average power (W)-mean ± SD	43.61 ± 13.34
Total energy (kJ)-median (IQR)	186.02 (114.97)
**Equipment error-n (%)**	
Device power cannot be turned on	0 (0.0)
No power output	0 (0.0)
No impedance reading	0 (0.0)
No RF connection	0 (0.0)
Cooling pump be stationary	0 (0.0)
No coolant flow or insufficient flow	0 (0.0)
Needle fracture	0 (0.0)
Power supply interruption	0 (0.0)
Needle bending-n (%)	7 (10.3)

IQR, interquartile range; RF, radiofrequency.

### Major adverse events and safety

All MAE are listed in Table 4. Most complications occurred during the intervention. Pericardial effusion/hemorrhage was noted in six patients (8.8%) during ablation or needle removal and was treated successfully with pericardiocentesis. There were no recurrences at follow-up. At routine examination 1 month after the procedure, one patient (1.5%) presented with pericardial effusion and was treated successfully with pericardiocentesis. None of the patients hat to undergo cardiac surgery. Nineteen patients (27.9%) experienced transient idioventricular rhythm during the procedure and recovered after a brief pause in ablation. One patient (1.5%) had complete left bundle branch block (CLBBB), and six patients (8.8%) had complete right bundle branch block (CRBBB). Their heart rhythm gradually returned to normal during subsequent ablations, and all the blocks were transient and resolved at the end of the procedure. None of the seven patients had a permanent heart block during follow-up. Ventricular fibrillation (VF) with hypotension occurred in one (1.5%) patient. The needle was removed, and the procedure was terminated prematurely. The patient underwent immediate cardiopulmonary resuscitation and VF could be terminated by defibrillation to sinus rhythm. Subsequently, the patient’s hemodynamics remained stable and without any neurologic deficit and this complication was without any further sequelae. Importantly, there were no MAE in this patient at 30-day, follow-up.

**TABLE 4:** Major adverse events within 30 days.

Group-n (%)	Device-related complications	Procedural complications
Pericardial effusion	6 (8.8)	1 (1.5)
Cardiac tamponade	0 (0.0)	0 (0.0)
Pleural effusion	0 (0.0)	0 (0.0)
Transient idioventricular rhythm	19 (27.9)	0 (0.0)
VT (> 120 bpm)	0 (0.0)	0 (0.0)
CLBBB	1 (1.5)	0 (0.0)
CRBBB	6 (8.8)	0 (0.0)
AVB	0 (0.0)	0 (0.0)
CHB	0 (0.0)	0 (0.0)
VF	1 (1.5)	0 (0.0)
Hypotension	1 (1.5)	0 (0.0)
Ventricular septal pseudoaneurysm	0 (0.0)	0 (0.0)
Ventricular septal perforation	0 (0.0)	0 (0.0)
Infection	0 (0.0)	0 (0.0)
Stroke	0 (0.0)	0 (0.0)
Recovery after SCD	1 (1.5)	0 (0.0)
Death	0 (0.0)	0 (0.0)

AVB, atrial ventricular block; CHB, complete heart block; CLBBB, complete left bundle branch block; CRBBB, complete right bundle branch block; SCD, sudden cardiac death; VF, ventricular fibrillation; VT, ventricular tachycardia.

### Effectiveness

**FIGURE 2:**
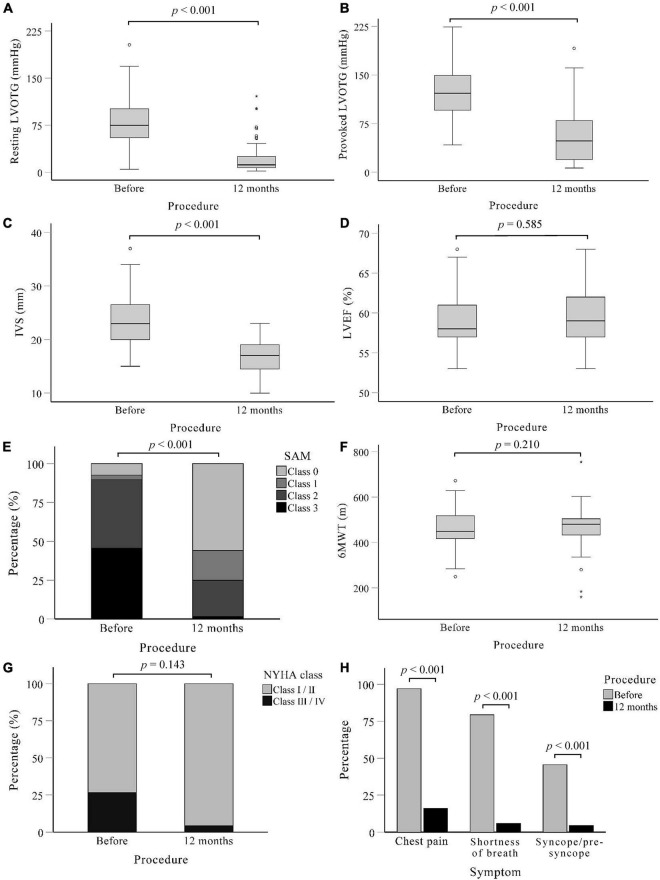
Post-procedure characteristics of patients. A systematic evaluation at 12 months after procedure showed that Liwen RF™ was effective in relieving outflow tract obstruction and symptoms compared to baseline. **(A)** Left ventricular outflow tract gradient under calm state. **(B)** Left ventricular outflow tract gradient under provocation of treadmill motion. **(C–E)** Measured in calm state. **(C)** Maximum thickness of interventricular septum. **(D)** Change in left ventricular ejection fractions. **(E)** Grades of mitral valve systolic anterior motion. **(F)** Results of the 6-min walk test. **(G)** The distribution of NYHA function class among patients. **(H)** Incidence of the three main symptoms of chest pain, shortness of breath, and syncope/pre-syncope. IVS, Interventricular septum; LVEF, Left ventricular outflow tract; LVOTG, Left ventricular outflow tract gradient; NYHA, New York heart association; SAM, systolic anterior motion; 6MWT, 6-min walk test.

Post-procedure characteristics are shown in Figure 2. The procedural success was 88.2% (60/68). At 12-months follow-up, the resting LVOT gradient was significantly lower than at baseline [75 (IQR: 48) *vs.* 12 (IQR: 19) mmHg, *p* < 0.001] and there was a significant reduction in the provoked LVOT gradient under physical exercise [122 (IQR: 53) *vs.* 41 (IQR: 59) mmHg, *p* < 0.001]. Two patients (2.9%) experienced paradoxical increases of resting and provoked LVOT gradients, and 1 patient (1.5%) had increases in provoked LVOT gradients. The maximum thickness of the IVS decreased from 23.56 ± 4.55 mm before the procedure to 16.53 ± 3.23 mm after the procedure (*p* < 0.001). Moreover, 17 patients (25.0%) presented with SAM (grade 2 and above) at rest after treatment, as compared to 61 patients (89.7%) before the procedure (*p* < 0.001). There were significant improvements in functional capacity: patients with New York Heart Association (NYHA) functional class ≥ III decreased from 18 (26.5%) patients at baseline to 3 (4.4%) at 12 months follow-up (*p* = 0.143). At the same time, the incidence of chest pain [66 (97.1%) *vs.* 11 (16.2%), *p* < 0.001], shortness of breath [54 (79.4%) *vs.* 11 (16.2%), *p* < 0.001] and syncope/pre-syncope [31 (45.6%) *vs.* 4 (5.9%), *p* < 0.001] all decreased at 12-month follow-up.

## Discussion

The Liwen RF ablation system has been evaluated in a small series of patients for the treatment of symptomatic HOCM which is resistant to medical management. In this study, a total of 68 patients who underwent septal reduction therapy with the Liwen technology between 2019 and 2020 were included. To our knowledge, this is the first, larger-scale study to report the application of conformal ablation in HOCM patients. This study shows that the application of the new system has been successful in all patients.

Current invasive treatment methods, SM and ASA, can relieve symptoms and improve prognosis by removing LVOT obstruction. It is worth noting that myectomy is an arduous challenge for patients, requiring thoracotomy and cardiopulmonary bypass with a heart-lung machine (27, 28), and the recovery time after the operation is quite long. Obstructions in the ventricular cavity and apex are often difficult to eliminate. In addition, the procedural success is dependent on the surgeon’s experience. ASA is less traumatic, compared to myectomy. However, the anatomical variation of the blood vessels supplying the IVS causes variable results regarding target ablation area and clinical results (gradient reduction) (29–31). Importantly, due to the presence of collateral vessels, the infarction area after alcohol injection may be larger than intended and can have serious consequences including large myocardial infarctions, complete atrial ventricular block (AVB) and/or sudden death (15). Endocardial radiofrequency ablation of septal hypertrophy (ERASH) is another transcatheter septal reduction therapy used as an alternative to SM and ASA intolerance (32, 33). It takes a vascular approach and reaches the myocardium through the endocardium. Despite successful LVOT gradient reduction and symptomatic improvement, current reports of ERASH complications and reduction of IVS thickness are inconsistent (34, 35). Given these outcomes, ERASH requires further study.

As for PIMSRA, the new device could change previous protocols for IVS ablation. In previous procedures, the choice of working section length is a delicate issue. Usually, one chooses between two types of needles (ablation needles with 1 or 2 cm electrode length). The longer the electrode is which is used for the procedure, the greater the energy output per unit time. A large area of the myocardium covered by ablation also means a reduced ability of control. Ventricular septal hypertrophy caused by HOCM is usually irregular and the site of hypertrophy may show great variation (36). Larger size ablation needles are often difficult to handle at thickened boundaries. To be safe, including protection of the conduction system and avoidance of septal perforation, we suggest that the smallest device possible should be used for the procedure, even if thicker LV parts may enable larger device sizes in certain LV areas. Because of this, the ablation may not be fully completed in one cycle, and it is often time-consuming and labor-intensive. The Liwen RF eliminates this hassle completely, with the ablation needle featuring a stepless adjustable electrode. With only one puncture, that is, without changing the needle type, it can handle IVS of different thickness in different parts. A matching ablation generator adjusts the output according to the electrode’s endurance and the changes in impedance resulting from the surrounding myocardial necrosis.

With the concept of conformal ablation, we conducted a preliminary trial using Liwen RF in 68 HOCM patients under the premise of ensuring safety. Three operators performed the procedure in this study. Prior to this, they performed a total of 126 PIMSRA procedures. Regarding safety, pericardial effusion or bleeding occurred mostly because of active hemorrhage of the IVS vessels from coronary vein injury. This apical approach results in small vessel injury and myocardial bleeding along the needle pathway, which is the main cause of pericardial effusion, thus attributable in part to learning curve and pre-procedural planning. The new insertion protocol considers pre-procedural CTA/MRI and intra-operative color Doppler flow imaging. Optimization of needle entry site and direction minimizes vascular injury. In all 68 patients, there was no cardiac tamponade or emergency sternotomy. The PIMSRA procedure uses a 17/18G needle, which remains intra-myocardial. Thus, the injury to myocardium from the needle is limited. In our experience, the use of adjustable needles on the Liwen RF under echocardiographic guidance can effectively reduce the incidence of bleeding. The ablation needle produces a local thermal effect through a high-frequency current. If the ablation boundary is too close to the endocardium, the subendocardial conduction system may be stimulated/injured by the RF energy. We speculate that this is a possible cause of conduction block, ventricular arrhythmias, and even VF. The arrhythmias we encountered were all temporary, thanks to accurate positioning and precise control of the ablation range (working section length). We encountered one patient in need of defibrillation for ventricular fibrillation, which taught us that we need a safe distance from the ablation boundary to the endocardium. There were no secondary abnormalities in the cardiac structure (ventricular septal perforation or ventricular septal pseudoaneurysm). All other patients had stable hemodynamics. We did not find any ablation system error including needle breakage, so we confirmed the stability of the system and the structural strength of the needle. At the same time, we observed that the needle was bent after removal in seven cases. These patients reported no adverse outcomes such as myocardial tears or bleeding. In our analysis, the flexion occurred *in vivo* at the costal margin rather than in the myocardium. The site of puncture and the degree of myocardial fibrosis may explain this phenomenon. Despite the excellent strength of the electrode needle, excessive bending is not recommended during use.

The 12-month follow-up provided insight into the effectiveness of the system. The procedure using Liwen RF can be considered effective. We observed an improvement in cardiac function after procedure. The incidence of chest pain, shortness of breath, and syncope/pre-syncope decreased significantly after LVOT obstruction was resolved, reflecting improvements in patients’ quality of life. Due to the increase of cardiac output and the improvement of systemic perfusion, the proportion of patients with NYHA function greater than class 2 decreased after the procedure. With the remission of symptoms, the 6-min walk distance also showed an increasing trend. On echocardiography, hemodynamic findings objectively improved. The absorption of necrotic tissue and myocardial remodeling after radiofrequency ablation reduced the maximum thickness of IVS. The LVOT gradient at rest decreased significantly consistent with a relief of obstruction. The LVOT gradient under provocation of treadmill exercise decreased after the procedure, indicating no obstruction of blood flow during a high dynamic state. The LVOT gradient reduction can diminish the anterior motion of the anterior mitral valve leaflet, thereby further improving the outflow tract gradient. The paradoxical increase in obstruction may be due to insufficient ablation and unintended cardiac remodeling. There was no significant change in LVEF because ventricular filling was limited before obstruction relief. Ventricular end-diastolic volume and stroke volume increased after the procedure. Due to limitations of the formula, the increased cardiac output was not reflected.

Although the procedures were performed with Liwen RF, we tend to attribute the results to the optimization of the needle rather than the procedure itself. The use of this stepless adjustable needle makes it possible for our operators to achieve conformal ablation. Since this is a study of the first-in-man application, it is not meaningful to compare Liwen RF and Cool-Tip, given the differences in treatment procedures between the two systems. We can expect that with the development of a new generation of Liwen RF ablation system, operators will be able to perform more complete and accurate ablations while avoiding complications as much as possible.

### Limitations

Patients with septal thickness of 30 mm or more were not included in our study. The safety and effectiveness of the device cannot be verified for use in this population. Subsequent upgrades or new models may remedy this deficiency.

Since this is a newly developed device, the accumulation of time spent by the operators and the learning curve of the doctors may have influenced the results. In addition, this was a preliminary study and the number of cases included in the study was limited. There may be rare complications and specific uses that have not been recognized. More conclusions about safety and applicability need to be answered by further multicenter, prospective studies. We were unable to compare the safety and efficacy of the two system. After the feasibility of the new device is confirmed, large-scale randomized controlled trials will be conducted to fill this gap.

## Conclusion

The Liwen RF is a novel device with a stepless adjustable electrode length. It is used to ablate hypertrophic IVS accurately and safely according to its irregular shape in the treatment of drug-resistant HOCM. For patients who cannot undergo SM or ASA, this device demonstrated a significant reduction in LVOT obstruction and alleviation of clinical symptoms during follow-up. The incidence of intraprocedural and postprocedural complications was low. This study demonstrates the feasibility of the Liwen RF ablation system for the treatment of drug-resistant HOCM.

## Data availability statement

The original contributions presented in the study are included in the article/supplementary material, further inquiries can be directed to the corresponding authors.

## Ethics statement

The studies involving human participants were reviewed and approved by the Ethics Committee of Xijing Hospital. The patients/participants provided their written informed consent to participate in this study.

## Author contributions

ZW drafted the article. ST, RH, and LL designed the study. HS, ST, SB, KP, and MZ revised the manuscript. ZW and RH designed figures. RZ, JnL, CL, XL, JaL, BX, and BF were responsible for patient management and data collection. All authors read and approved the final manuscript.

## Abbreviations

ASA: Alcohol septal ablation
HOCM: Hypertrophic obstructive cardiomyopathy
IVS: Interventricular septum
LVEF: Left ventricular ejection fractions
LVOT: Left ventricular outflow tract
MAE: Major adverse events
NYHA: New York Heart Association
PIMSRA: Percutaneous intramyocardial septal radiofrequency ablation
SAM: Systolic anterior motion
SCDI: Sudden cardiac death index
SM: Surgical myectomy
TTE: Transthoracic echocardiography
VF: Ventricular fibrillation.
